# Automatic screening and classification of diabetic retinopathy and maculopathy using fuzzy image processing

**DOI:** 10.1007/s40708-016-0045-3

**Published:** 2016-03-16

**Authors:** Sarni Suhaila Rahim, Vasile Palade, James Shuttleworth, Chrisina Jayne

**Affiliations:** 1 Faculty of Engineering, Environment and Computing, Coventry University, Priory Street, Coventry, CV1 5FB UK; 2 Faculty of Information and Communication Technology, Universiti Teknikal Malaysia Melaka, Hang Tuah Jaya, 76100 Durian Tunggal, Melaka Malaysia

**Keywords:** Diabetic retinopathy, Maculopathy, Eye screening, Colour fundus images, Fuzzy image processing

## Abstract

Digital retinal imaging is a challenging screening method for which effective, robust and cost-effective approaches are still to be developed. Regular screening for diabetic retinopathy and diabetic maculopathy diseases is necessary in order to identify the group at risk of visual impairment. This paper presents a novel automatic detection of diabetic retinopathy and maculopathy in eye fundus images by employing fuzzy image processing techniques. The paper first introduces the existing systems for diabetic retinopathy screening, with an emphasis on the maculopathy detection methods. The proposed medical decision support system consists of four parts, namely: image acquisition, image preprocessing including four retinal structures localisation, feature extraction and the classification of diabetic retinopathy and maculopathy. A combination of fuzzy image processing techniques, the Circular Hough Transform and several feature extraction methods are implemented in the proposed system. The paper also presents a novel technique for the macula region localisation in order to detect the maculopathy. In addition to the proposed detection system, the paper highlights a novel online dataset and it presents the dataset collection, the expert diagnosis process and the advantages of our online database compared to other public eye fundus image databases for diabetic retinopathy purposes.

## Introduction

Diabetic retinopathy is one of the eye complications caused by the diabetes mellitus, which causes other problems such as stroke, cardiovascular disease, diabetic nephropathy and diabetic neuropathy. Diabetes mellitus produces damage to retinal capillaries and it can be visualised only in the retina, a transparent tissue of several different layers of cells [1]. Diabetic retinopathy results in visual disturbances and can lead to permanent blindness. Therefore, an effective diabetic retinopathy screening is essential for early treatment, along with an effective risk factor management to prevent diabetic complications and reduce morbidity and mortality impact.

In order to perform a screening of diabetic retinopathy, there are various tools available, such as the direct ophthalmoscope, PAN ophthalmoscope, binocular indirect ophthalmoscope, slit lamp and fundus camera [2]. There are two types of fundus cameras. The first type is mydriatic, where the pupil dilation is required. The second type is non-mydriatic, which is easy to use, patient-friendly and pupil dilation is done only if necessary. The fundus camera provides high quality of digital photographs, where the fundus photos can be instantly viewed and shown to the patients to increase the patient’s understanding of the disease [2].

Microaneurysms, the earliest visible signs of diabetic retinopathy, appear as small red dots on the retina. There are other diabetic retinopathy signs, such as haemorrhages, i.e. red lesions caused by the rupture of the small blood vessels in the deeper layers of the retina. Exudates, which are yellow–white lesions caused by plasma leakage from the capillaries, are another type of common features of diabetic retinopathy. However, if the exudates are found within one disc diameter (1DD) of the fovea, they are called exudative maculopathy.

**Fig. 1:**
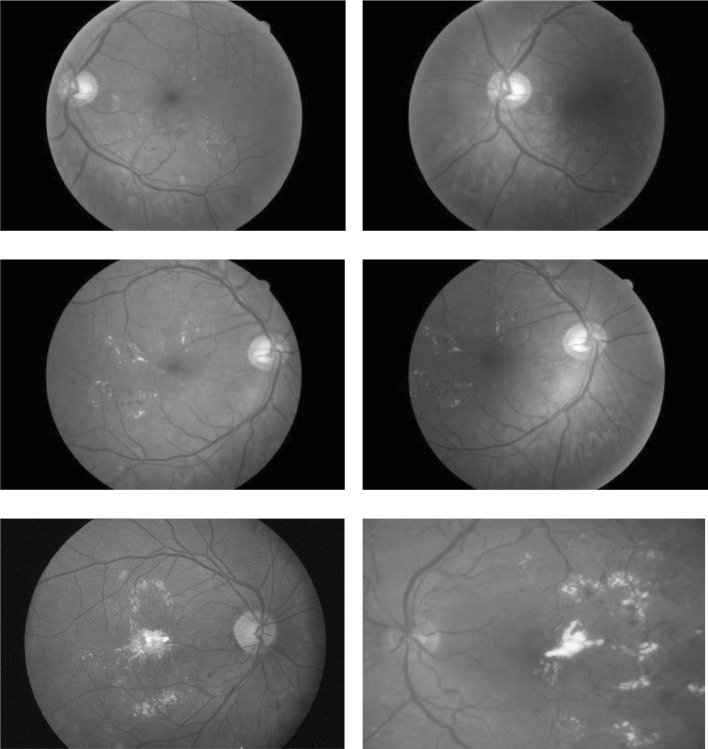
Diabetic maculopathy stages [55]. Mild maculopathy. Moderate maculopathy. Severe maculopathy

In addition to the diabetic retinopathy signs detection, the maculopathy detection is also important in order to indicate the urgency of the referral based on the presence or absence of the maculopathy [3]. Diabetic maculopathy affects the visual function through the macular ischaemia and increased retinal vascular permeability resulting in macular oedema [3]. Maculopathy is represented by yellow lesions near the macula region and it is the diabetes damage near the fovea. The centre of the macular region is a tiny area called the fovea, where all the detailed vision is provided and it represents the most sensitive area of the retina. So, the lesions in this area are of particular concern, as they can cause loss of vision. It is therefore vital to have a screening and grading of all lesions in the macular region as it affects the visual acuity as well as other visible abnormalities presence. The leakage of capillary in the macular region is associated with macular oedema, i.e., the swelling of the retina, and it affects sight. The macula oedema is not visible but can cause damage to the fovea [1]. Figure 1 shows the fundus images for the diabetic maculopathy different stages. The classification into different severity levels of maculopathy, i.e., mild, moderate and severe levels, are based on the location of exudates in macula region.

Fuzzy image processing uses fuzzy techniques in the various stages of an image processing task. Fuzzy techniques can help not only to produce better representation of images and improve the performance analysis but also can help produce a more reliable screening system. Fuzzy image processing has been reported to work well as fuzzy histogram equalisation method by Sheet et al. [4], Garud et al. [5] and Rahim et al. [6–8]. Fuzzy filtering is another fuzzy image processing method, proposed by Patil and Chaudhari [9], Toh et al. [10, 11], Kwan [12, 13] and also Rahim et al. [7, 8]. In addition, fuzzy edge detection can be used as a preprocessing technique in order to enhance the image quality and is reported in [7, 8].

The paper is organised as follows. Section 2 presents previous related work on automatic methods for diabetic retinopathy and maculopathy detection. Section 3 describes in details the novel dataset developed by us, while Sect. 4 presents the proposed system for the detection of diabetic retinopathy and maculopathy in eye fundus images by using fuzzy image processing. Finally, Sect. 5 presents some conclusions and future work.

## Previous related work

Several systems for detection and diagnosis of diabetic retinopathy have been reported in the literature [14–17]. Some of the reported developments focus and propose techniques for detecting certain features of diabetic retinopathy, such as microaneurysms in [18–23], exudates in [24–28], haemorrhages in [27, 29] and neovascularisation in [28, 30–32].

In our earlier work, we have proposed a preliminary system for the detection of diabetic retinopathy by using a combination of non-fuzzy techniques [33]. We have also presented several individual systems for the automatic detection of microaneurysms in colour fundus images for diabetic retinopathy screening in [6]. The first system highlights the automatic detection of microaneurysms in colour fundus images using segmentation and feature extraction. We have proposed two subsystems for an automatic detection of microaneurysms in colour fundus images using the Circular Hough Transform method for the localisation of the microaneurysms, due to the capability of this method to detect circular shapes. The first subsystem proposed a combination of image processing techniques and circular Hough transform, while the second subsystem presented the detection of microaneurysms using fuzzy image processing. The first system, which applies a non-fuzzy technique for image preprocessing is compared with the second system, which implements a fuzzy image preprocessing technique. Based on the results obtained, it can be concluded that the implementation of fuzzy preprocessing techniques provide better contrast enhancement for fundus images and it greatly assists in detecting the microaneurysms.

Due to the promising results in the implementation of fuzzy histogram equalisation technique for the detection of microaneurysms, we enhanced the development of the microaneurysms detection system by proposing a novel dataset and other fuzzy techniques in the image preprocessing part. We proposed the implementation of fuzzy filtering and fuzzy edge detection separately, in addition to the fuzzy histogram equalisation as mentioned before, for the automatic detection of microaneurysms [7]. The fuzzy techniques work better for fuzzy histogram equalisation and fuzzy edge detection. The analysis shows that the implementation of the fuzzy preprocessing techniques provides better contrast enhancement and other improvements such as brightness and better segmentation for fundus images. The use of fuzzy image processing techniques plays an important role in producing better image quality and improved performance analysis. We investigated the capability of a combination of different fuzzy image processing techniques for the detection of diabetic retinopathy and maculopathy in eye fundus images in [8]. The proposed system implements a combination of fuzzy techniques in the image preprocessing part, which combine fuzzy filtering, followed by the fuzzy histogram equalisation and fuzzy edge detection. The system first classified images into two classes, and then into ten classes, which provide more details about the stage of the disease. The results show that the two-class classifiers identify much better the diabetic retinopathy cases compared to the ten classes’ case. This is due to the fact that there were more images in the two-class dataset compared to the ten classes’ case (when some of the ten classes were not represented by enough images in the dataset). In addition, the result shows that the maculopathy can be seen clearly from the generated output image.

### Fuzzy image processing for medical images

Fuzzy approaches are widely implemented in image processing system developments reported in the literature, mainly for non-medical images but, in a smaller number of cases, for medical image processing too. This section highlights the use of fuzzy approaches performed particularly in the processing stage on medical images. The fuzzy image processing techniques that can be implemented are fuzzy filtering, fuzzy contrast enhancement, fuzzy image segmentation and fuzzy edge detection.

Li et al. [34] proposed a novel fuzzy level set algorithm for medical image segmentation based on the segmentation obtained by spatial fuzzy clustering. The proposed algorithm leads to a more robust segmentation and effectiveness for medical image segmentation tasks. Moreover, for fuzzy contrast enhancement, Sheet et al. [4] proposed a novel modification of the brightness preserving dynamic histogram equalisation, called the Brightness Preserving Dynamic Fuzzy Histogram Equalisation (BPDFHE), in order to improve the brightness preservation and contrast enhancement capabilities, but at the same time reducing its computational complexity. The proposed technique has been tested later by Garud et al. in [5] to investigate the ability of the technique for digital pathology images. The results show that the proposed technique can preserve the image brightness better than histogram equalisation and techniques based on contrast-limited adaptive histogram equalisation. In addition, fuzzy filter techniques proposed in [10–13] aim to detect and remove the noise from the corrupted image.

### Maculopathy detection methods

In order to identify the maculopathy, the localisation and the detection of macula and fovea are essential, as the maculopathy is represented by lesions in the macula region and fovea is the centre of macula. Kumar et al. [35] proposed an approach in detecting the macula by using bit plane decomposition and mathematical morphology methods. Mubbashar et al. [36] presented an automated system for the localisation and detection of macula in digital retinal images. The optic disc centre and blood vessels extraction are performed prior to the detection of the macula by using the centre of optic disc and thresholding, followed by vessel enhancement and locating the macula as the darkest pixels in the region. In addition, the detection of macula is proposed by Akram et al. [37] along with the detection of exudates. The system proposed a contrast enhancement, thresholding and blood vessel segmentation to detect the dark candidate, followed by the classification of some features set for the macula detection by using a Gaussian mixtures model-based classifier. The localisation of the optic disc and the fovea in retinal fundus images is also proposed by Sekhar et al. [38]. Morphological operations and Hough transform are implemented to locate the optic disc, while the fovea is located by using the spatial relationship with the optic disc and from the spatial distribution of the macula. They defined the region of interest as an area of a sector originating at the optic disc centre by an angle of 30° above and below the line between the optic disc centre and the centre of the retinal image disc.

The detection of maculopathy is vital as it will eventually cause loss of vision if the affected macula is not timely treated. Therefore, some researchers are focusing on this challenging area and suggest several solutions on the detection of the maculopathy on the retinal images. Punnolil [39] proposed an approach for the diabetic maculopathy grading by implementing the detection of the retinal structures, such as optic disc, macula and fovea followed by the detection of the lesions including the exudates, haemorrhages and microaneurysms. These were later graded into classes of diabetic maculopathy using a multiclass Support Vector Machines (SVM) classifier based on the extracted features. Tariq et al. [40] present a similar diabetic maculopathy grading system starting with the optic disc localisation and vascular structure to extract the macula. Next, some features, such as area, compactness, mean intensity, mean hue and others, are extracted from the exudates detection and used to classify into different stages of maculopathy by using a Gaussian Mixture Model-based classifier. The detection of diabetic maculopathy in retinal images is also investigated by Vimala and Kajamohideen [41] by using morphological operations. The detection of macula is generated from the image preprocessing techniques, such as green component extraction, filtering and adaptive histogram equalisation. Next, morphological operations, such as top-hat transform and bottom-hat transform, are performed. Other automatic grading of diabetic maculopathy systems are proposed by Siddalingaswamy and Prabu [42], Hunter et al. [43] and Chowriappa et al. [44]. Siddalingaswamy and Prabu [42] proposed the detection of the optic disc, fovea and macula region, based on the location and diameter of the optic disc, followed by the detection of the hard exudates using clustering and mathematical morphological techniques. The classification into severity levels of maculopathy, which consists of mild, moderate and severe levels, is performed based on the location of exudates in marked macular region. An automated diagnosis of maculopathy system is presented by Hunter et al. [43]. First the optic nerve head and fovea are detected in order to locate the macula region. Next, candidate lesions are segmented, followed by feature extraction and, finally, the classification by a multilayer perceptron. An ensemble selection for feature-based classification of diabetic maculopathy images is suggested by Chowriappa et al. [44] based on extracting textural features and then classifying into the disease severity classes by using classifiers such as the hidden Naïve Bayes, Naïve Bayes, sequential minimal optimisation (SMO) and the tree-based J48 algorithm.

However, fuzzy processing has not been implemented during the preprocessing stage within these previously reported maculopathy detection systems. Therefore, this proposed system implements a combination of fuzzy techniques for the image processing part for the diabetic retinopathy and maculopathy detection.

## Experimental datasets

Some of the most popular public databases which contain the eye fundus images are the Standard Diabetic Retinopathy Database Calibration Level 0 (DIARETDB0), the Standard Diabetic Retinopathy Database Calibration Level 1 (DIARETDB1), Methods to Evaluate Segmentation and Indexing techniques in the field of Retinal Ophthalmology (MESSIDOR), Digital Retinal Images for Vessel Extraction (DRIVE), STructured Analysis of the Retina (STARE), Retinal Vessel Image set for Estimation of Widths (REVIEW) and the Retinopathy Online Challenge (ROC) database.

### Existing datasets

The DIARETDB0 dataset consists of 130 colour fundus images of which 20 are normal and the remaining 110 contain signs of diabetic retinopathy, such as hard exudates, soft exudates, microaneurysms, haemorrhages and neovascularisation. The original images, which are of size 1500 × 1152 in PNG format, are captured with 50° field-of-view digital fundus cameras with unknown camera settings [45]. In addition to the DIARETDB0, there is another dataset developed by the Machine Vision and Pattern Recognition Research Group, at Lappeenranta University of Technology, Finland, which is DIARETDB1, with 89 colour fundus images, combining 84 images that contain at least mild non-proliferative signs (microaneurysms) of diabetic retinopathy and five normal images [46]. The MESSIDOR database is another dataset produced by the research funded by the French Ministry of Research and Defence to facilitate studies on diabetic retinopathy diagnosis [47]. It consists of 1200 colour fundus images, captured using a colour video 3CCD camera on a Topcon TRC NW6 non-mydriatic retinography with a 45° field of view. The images acquired by three ophthalmologic departments have three different sizes: 1440 × 960, 2240 × 1488 and 2304 × 1536 pixels and also 8 bits colour plane [47]. Another database with retinal images is from the DRIVE project [48], which offers retinal colour images and results of automatic segmentation of blood vessels. The set of 40 images, where 33 do not show any sign of diabetic retinopathy and seven show signs of mild early diabetic retinopathy, were captured using a Canon CR5 non-mydriatic 3CCD camera with a 45° field of view, 8 bits per colour plane and of size 768 by 584 pixels [48].

The STARE project by Dr. Michael Goldbaum at the University of California, San Diego is funded by the U.S. National Institutes of Health produced another database with retinal colour images [49]. The set of 400 raw images with the list of the diagnosis codes and the diagnosis for each image can be obtained from the STARE database. Blood vessel segmentation work involves 40 of these images [49], while 80 images are used for optic nerve detection [50]. The DRIVE and STARE dataset are excellent databases of retinal vessel pixel segmentations; however, they do not include width measurements. Thus, the REVIEW [51] dataset is presented to fill this gap. The dataset includes 16 images with 193 vessel segments and a variety of pathologies and vessel types. The database contains accurate width measurements and four subsets of images, which are categorised in four classes: high resolution, vascular disease, central light reflex and kick points. Al-Diri and others [51] reported about the REVIEW dataset for retinal vessel and the algorithm used to process the segmentation to produce vessel profiles. The ROC presents an online competition for numerous methods for microaneurysms detection to compare with each other on the same dataset [52]. The images have three different sizes: 768 × 576, 1058 × 1061 and 1389 × 1383. The dataset consists of 50 training images of colour fundus photographs with available reference standard, and 50 test images where the reference standard was withheld by the organisers.

In addition to the public datasets presented above, a combination of normal and Diabetic Retinopathy (DR) fundus images from a novel dataset was developed as part of this project. The fundus images were collected from the Eye Clinic, Department of Ophthalmology, Hospital Melaka, Malaysia. The details of the developed dataset are presented in [7], under Sect. 4—“Proposed System”.

### Novel developed dataset

**Fig. 2:**
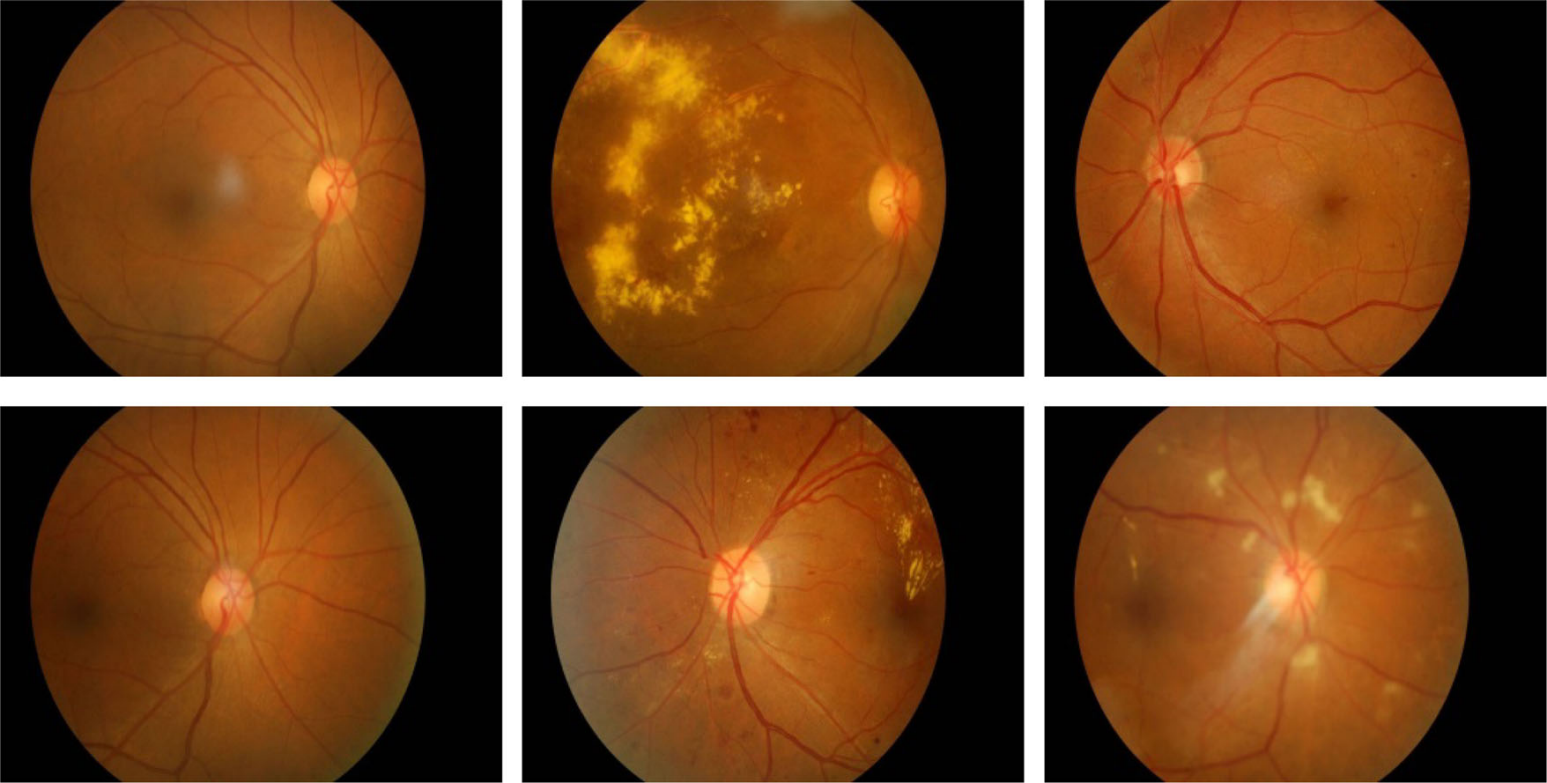
Examples of images in the dataset. Macula centre. Optic disc centre

**Fig. 3:**
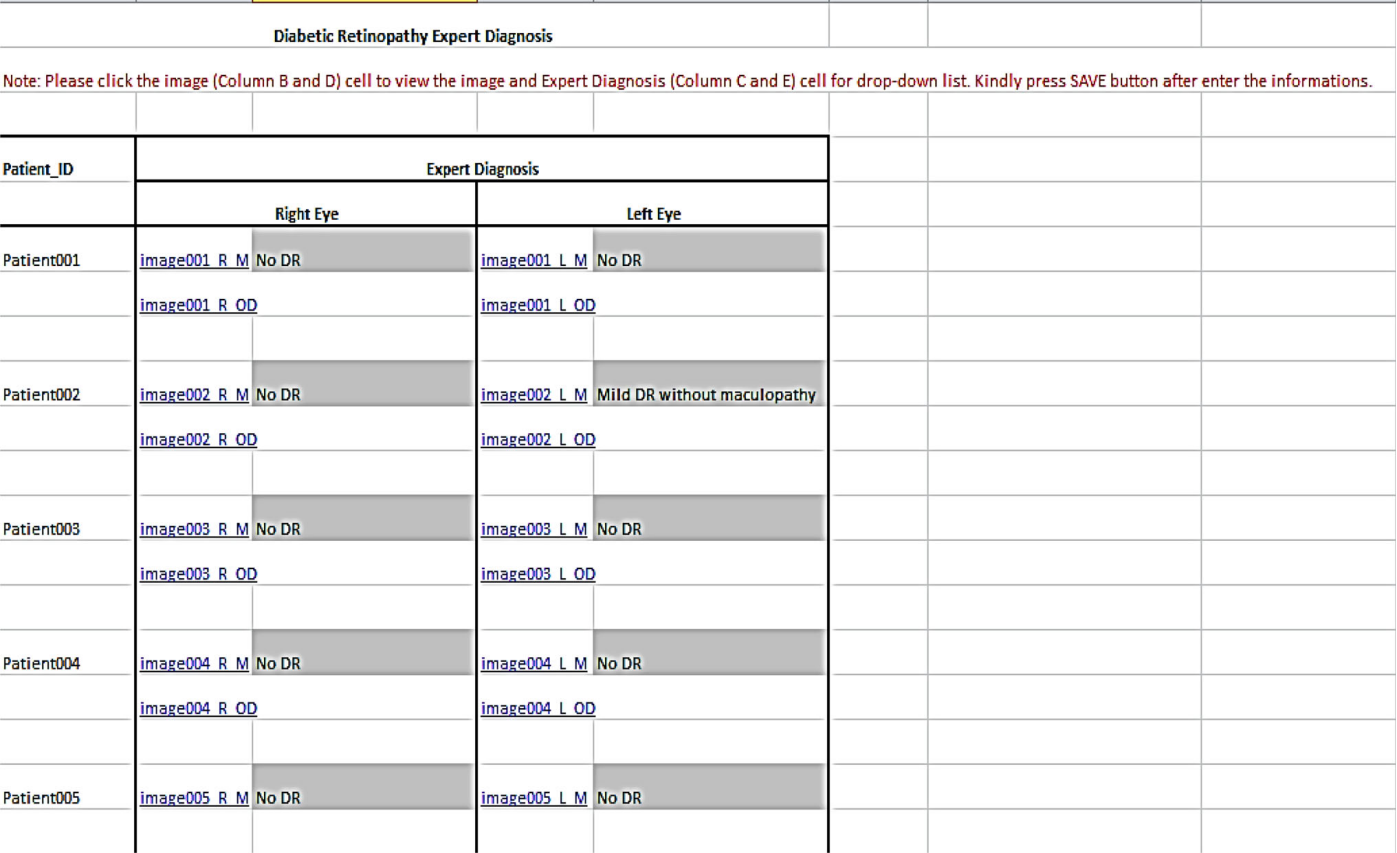
Expert diagnosis file

**Fig. 4:**
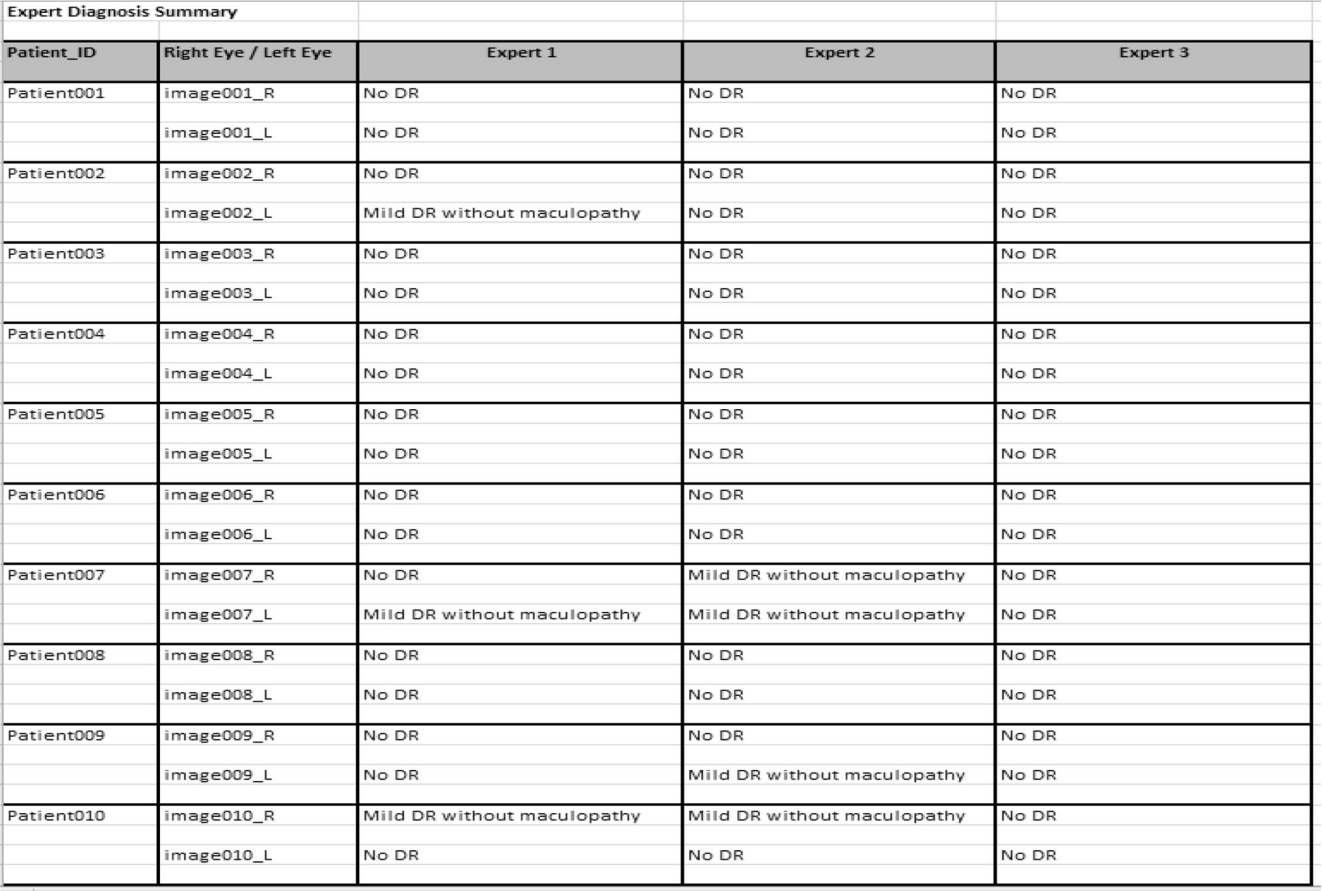
Summary of experts’ diagnosis

**Fig. 5:**
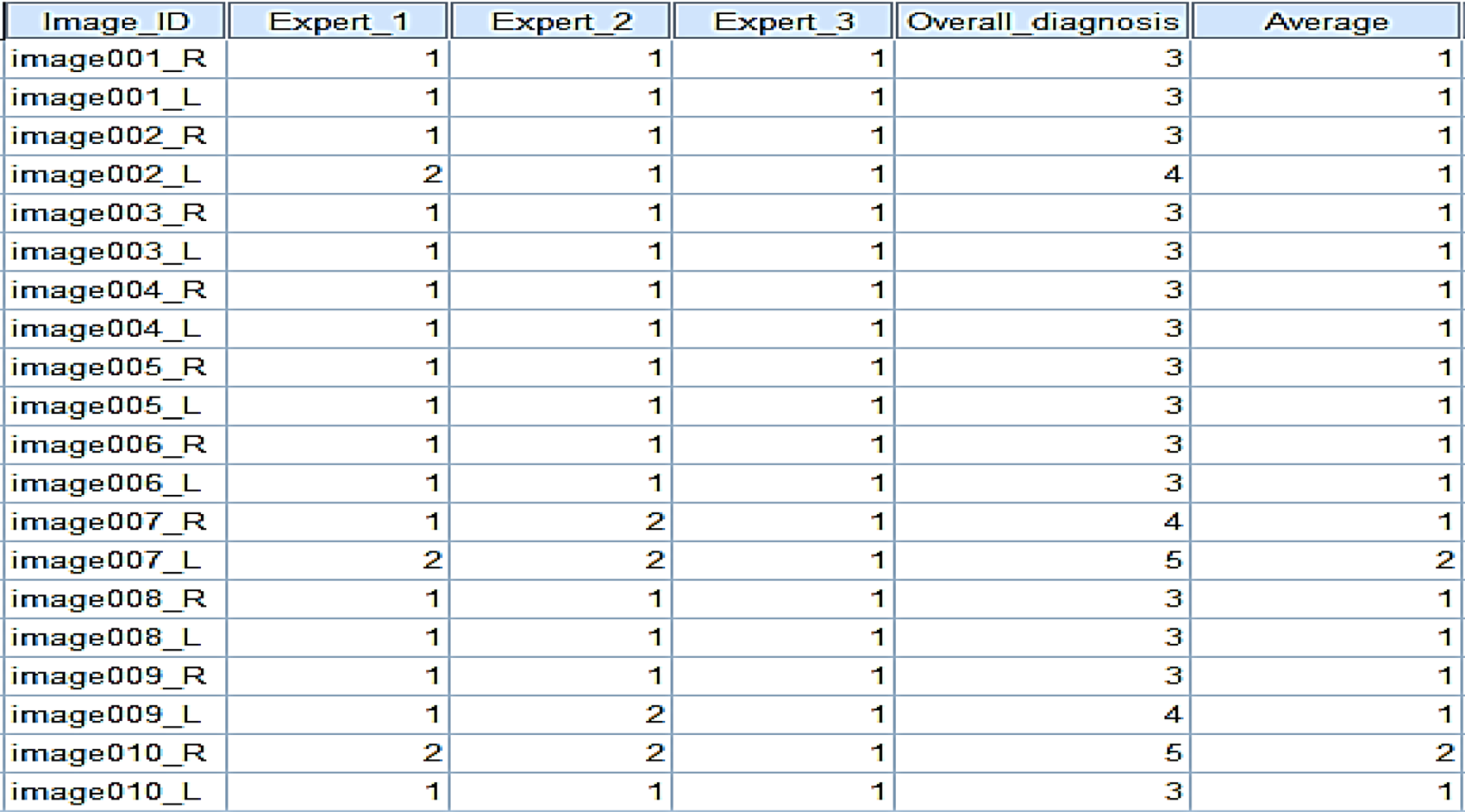
Averaging the experts diagnosis

The novel dataset consists of 600 colour fundus images from 300 patient’s folders collected at the Hospital Melaka, Malaysia. Each of the patient’s folders has two images minimum, at least one for the right side eye and one for the left side, where two different angles were captured, with optic disc centre and macula centre, respectively. The original images, which are of size 3872 × 2592 in JPEG format, provide high-quality details. These were captured with a KOWA VX-10 digital fundus camera. Figure 2 shows some examples of the images from the new developed dataset. Three experts from the Department of Ophthalmology, Hospital Melaka, Malaysia were involved to diagnose the fundus images into ten retinopathy stages: No Diabetic Retinopathy, Mild DR without maculopathy, Mild DR with maculopathy, Moderate DR without maculopathy, Moderate DR with maculopathy, Severe DR without maculopathy, Severe DR with maculopathy, Proliferative DR without maculopathy, Proliferative DR with maculopathy and Advanced Diabetic Eye Disease (ADED). An excel file containing the link to each eye fundus image and the retinopathy stages drop down list, as presented in Fig. 3, was provided to each of the three experts separately to avoid bias. The summary findings of the three experts are presented in Fig. 4. The average from the three experts is used for the overall expert diagnosis, as shown in Fig. 5.

As a result of the analysis on the experts’ diagnosis performed by using the SPSS software, the total number of images in each class is as follows: normal (no retinopathy) class with 276 images, while the abnormal or diabetic retinopathy (DR) class can be divided into nine other categories: mild DR without maculopathy (72), mild DR with maculopathy (27), moderate DR without maculopathy (85), moderate DR with maculopathy (83), severe DR without maculopathy (23), severe DR with maculopathy (11), proliferative DR without maculopathy (6), proliferative DR with maculopathy (10) and, finally, advanced diabetic eye disease, ADED (7). These are presented in Table 1.

**Table 1:** Expert diagnosis summary

Retinopathy Stage	No. of Images
No DR	276
Mild DR without maculopathy	72
Mild DR with maculopathy	27
Moderate DR without maculopathy	85
Moderate DR with maculopathy	83
Severe DR without maculopathy	23
Severe DR with maculopathy	11
PDR without maculopathy	6
PDR with maculopathy	10
ADED	7
Total	600

**Fig. 6:**
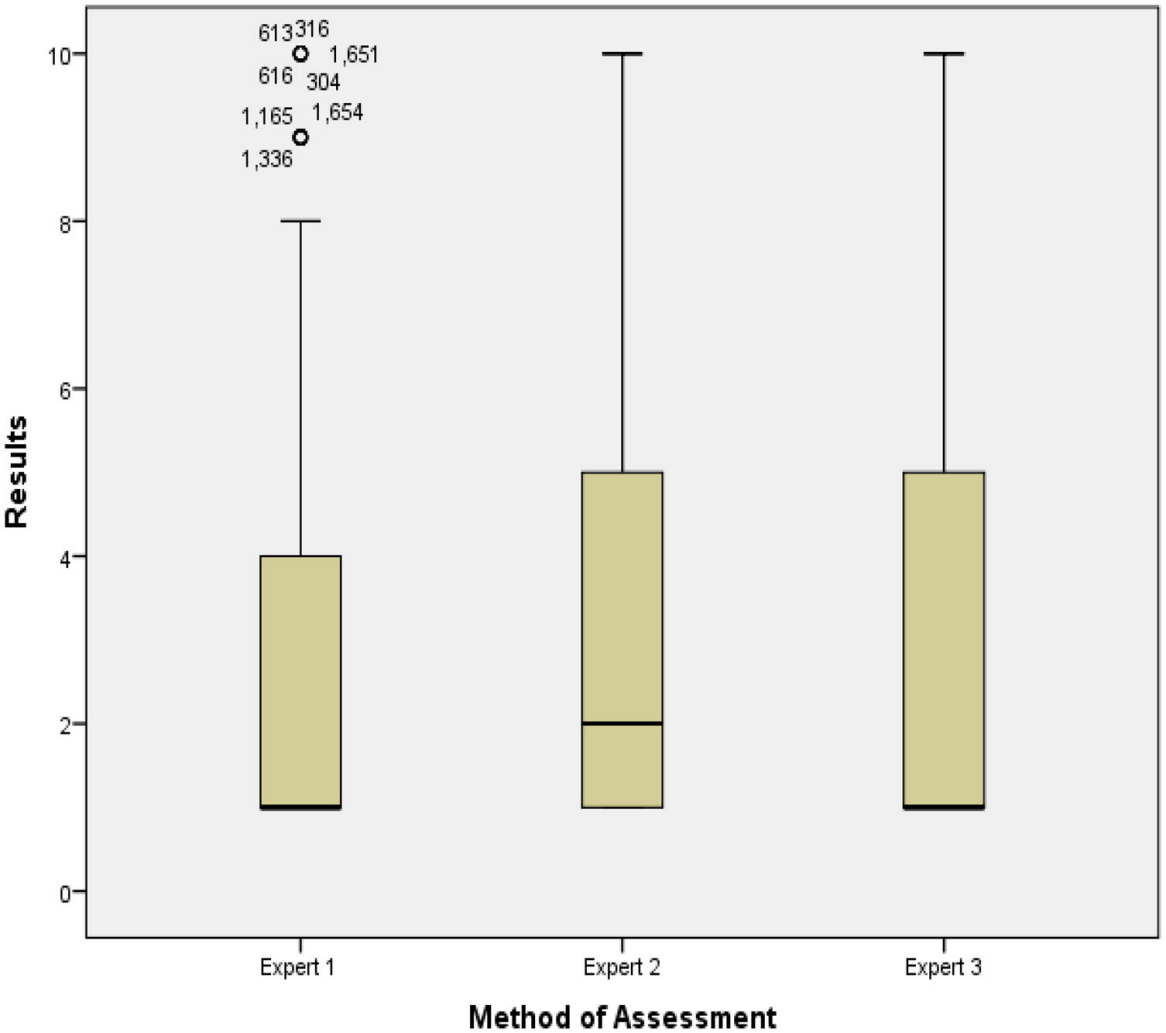
Boxplot assessment

Based on the ground truth provided by the experts, several descriptive and inferential analysis tasks using the SPSS statistical package were performed. The boxplot is the first method of assessment used for the descriptive analysis. It is a useful visualisation for viewing how the data are distributed. In addition, the boxplot is capable to display the distribution of scale variable and pinpointing outliers. Moreover, the boxplot shows the five statistics which are minimum, first quartile, median, third quartile and maximum value. Figure 6 shows the representation of the boxplot performed on the ground truth, which involves a total of 1800 images, i.e., 600 from each expert. It shows that for the first expert, there are outliers; the extreme values, which do not fall in the inner fences. The outliers mean that the values are not frequent values, particularly for the first expert, and, most of the time, these values are not within the normal range. Meanwhile, the dark line shows the median, which is the measure of central tendency. It shows that for the first and third experts, the median value is 1 (no diabetic retinopathy), while for the second expert, the median generated is 2 (mild diabetic retinopathy without maculopathy). The boxplot also shows the 25th percentile, representing 25 % of cases/rows that have values below the 25th percentile, in addition to the 50 % of the case/row that lie within the box and 75th percentile.

**Fig. 7:**
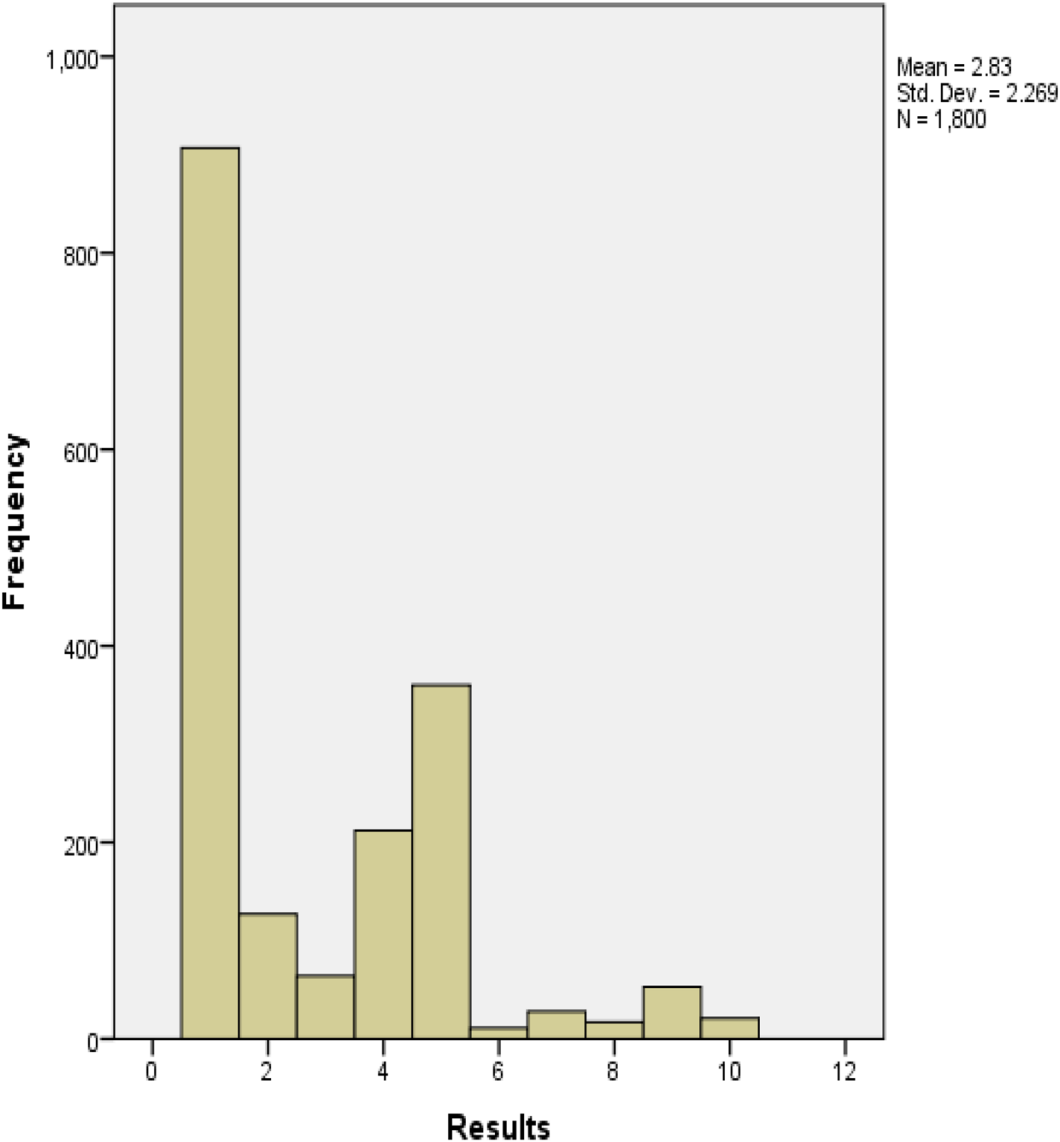
Histogram assessment

Using histograms is an alternative for a descriptive analysis that can be performed for this application. It is a visual summary of the distribution of values and it is useful for showing the distribution of a single-scale variable. Figure 7 shows the representation of the histogram, where the 1800 images from the three experts are binned into the ten retinopathy stages. It shows that the majority of the images are classified into the first stage, which is no retinopathy, followed by the fifth stage which is moderate diabetic retinopathy with maculopathy. The histogram also generated the mean (2.83) and the standard deviation (2.269) for the 1800 images used for the ground truth.

Analysis of variance (ANOVA) test is an inferential analysis that can be performed on the expert diagnosis ground truth. The test produces a one-way analysis of variance for a quantitative dependent variable by a single-factor (independent) variable. The analysis of variance is used to test the hypothesis that several means are equal. The test generated shows a *p*-value of 0.000. Next, the post hoc test is performed to determine which means are different. Table 2 shows the output of the post hoc test showing the multiple comparisons of the experts’ means. It can be concluded from Table 2, that there are differences between the first expert and the second and third expert; however, there is similarity between the second and the third expert.

**Table 2:** ANOVA multiple comparisons

Method of Assessment		*p*-value
Expert 1	Expert 2	0.001
	Expert 3	0.001
Expert 2	Expert 1	0.001
	Expert 3	1.000
Expert 3	Expert 1	0.001
	Expert 2	1.000

Chi-square test tabulates a variable into categories and computes a Chi-square statistic. It compares the observed and expected frequencies in each category to test that all categories contain the same proportion of values, or it tests that each category contains a user-specified proportion of values. In this case, the Chi-square is useful for determining if there is a relationship among the experts. Table 3 shows the cross tabulation results of the Chi-square test. The inferential statistic with the *p*-value of 0.000 indicates there is no relationship among the three experts. Table 3 represents the number of images in each category for the three experts alongside the percentage.

**Table 3:** Chi-square analysis

Results	Method of assessment					
	Expert 1		Expert 2		Expert 3	
	Count	%	Count	%	Count	%
No DR	326	54.3	267	44.5	314	52.3
Mild DR without maculopathy	55	9.2	58	9.7	14	2.3
Mild DR with maculopathy	21	3.5	12	2.0	31	5.2
Moderate DR without maculopathy	79	13.2	90	15.0	43	7.2
Moderate DR with maculopathy	90	15.0	127	21.2	143	23.8
Severe DR without maculopathy	5	0.8	6	1.0	0	0.0
Severe DR with maculopathy	3	0.5	6	1.0	19	3.2
PDR without maculopathy	6	1.0	9	1.5	2	0.3
PDR with maculopathy	8	1.3	20	3.3	25	4.2
ADED	7	1.2	5	0.8	9	1.5
Total	600	100	600	100	600	100
*p*-value	0.000					

Furthermore, the ground truth provided by the experts can be categorised into three categories. The variety of the categorisation, shown in Table 4, can be used for testing and, as a result, various analyses can be performed. The first categorisation divides the images into two classes, which are no diabetic retinopathy and diabetic retinopathy, while the second categorisation consists of four stages, involving no diabetic retinopathy stage, non-proliferative diabetic retinopathy (mild, moderate and severe cases), proliferative diabetic retinopathy and advanced diabetic eye disease. Finally, the third categorisation is the one that contains the ten retinopathy stages.

**Table 4:** Expert diagnosis summary categorisation

Categorisation I		Categorisation II		Categorisation III	
No DR	276	No DR	276	No DR	276
DR	324	Non-proliferative DR	301	Mild DR without maculopathy	72
		Proliferative DR	16	Mild DR with maculopathy	27
		ADED	7	Moderate DR without maculopathy	85
				Moderate DR with maculopathy	83
				Severe DR without maculopathy	23
				Severe DR with maculopathy	11
				PDR without maculopathy	6
				PDR with maculopathy	10
				ADED	7
Total	600		600		600

The new dataset is different compared to other datasets which are presented earlier in Sect. 3.1. The dataset represents South East Asian population, particularly Malaysian, compared to the other datasets, which represent Caucasian population. It provides almost a balanced total number of No DR/Normal and DR/Abnormal images. Typically, in medical images diagnosis, it is difficult to find a large number of normal cases. The imbalanced number of available images poses some problems in the classification phase; therefore, the balanced number in our dataset will help overcome this problem.

Moreover, the categorisation of the expert diagnosis followed the standard practice based on the International Clinical Retinopathy and Diabetic Macula Oedema Disease Severity Scale. The classification of data involves maculopathy, which is the yellow lesion near the macula. This is a very detailed categorisation compared to other datasets. The detection of maculopathy is very important, as the macula is responsible for central vision and it represents a sensitive part of the eye and hence it is vital for detecting the urgency of the referral. In addition, the novel dataset and the expert diagnosis may be used for the diabetic retinopathy grading as well as for the diabetic maculopathy grading, separately.

In order to make the novel fundus images dataset widely accessible, the dataset has been made available as an online database. The novel dataset is accessible at http://creative.coventry.ac.uk/fundus. The webpage of this research contains the novel dataset with eye fundus images, including the expert diagnosis file and the published papers related to this research project. The aim of this online database is to highlight the research project development and to promote research on retinal imaging to enable comparative studies and, most importantly, to share the eye fundus images with other researchers. The dataset can be downloaded for research and educational purposes.

## Proposed system: detection of diabetic retinopathy and maculopathy in eye fundus images using fuzzy image processing

The proposed system implements a combination of fuzzy techniques for the image preprocessing part, such as fuzzy filtering and fuzzy histogram equalisation. In addition, the proposed system implements the localisation and the detection of four retinal structures, which are the optic disc, the blood vessels, the macula and the fovea, which are important in the identification of the maculopathy. Furthermore, the system detects the diabetic retinopathy lesions, the exudates, which are vital in order to detect the exudative maculopathy. Several features extracted from the exudates lesions and the maculopathy are used for the classification. The system is evaluated with the combination of normal and diabetic retinopathy images, including the maculopathy fundus images from a novel dataset developed as mentioned above in the Sect. 3.2. The proposed system builds on the systems proposed in [7] and [8]. The previous system in [7] implements the use of fuzzy techniques for the image processing stage, including the fuzzy histogram equalisation, fuzzy median filter and fuzzy edge detection, for the purpose of one of the important lesion detection in diabetic retinopathy screening—microaneurysms. However, the fuzzy techniques are implemented individually in different variants of the system. On the other hand, the system in [8] proposed the implementation of the combination of several fuzzy image preprocessing techniques for the detection of the maculopathy without the retinal structures localisation. Three features, which are the area of on pixels, the mean and the standard deviation of the white pixels area, are extracted from the output image for the classification stage, which classifies into two main categories with the aid of several machine learning classifiers. Due to the promising results of both systems reported in [7] and [8], especially on the fuzzy techniques ability, we continued our developments with the proposed system to investigate the capability of the combination of the fuzzy image processing techniques, together with retinal structures detection approach and by using more extracted features, in order to produce a more reliable system for the detection of diabetic maculopathy.

The proposed diabetic retinopathy and maculopathy detection system has been created using Matlab R2014a environment. The system starts with the image acquisition process, where the system selects images from the image's folder for further processing. Next, the image preprocessing task takes place in order to improve the image quality. This task includes the implementation of the fuzzy image processing techniques and the retinal structures’ localisation and segmentation. In order to detect the maculopathy, the lesions called exudates have to be identified, where exudates situated in the macula region represent the exudative maculopathy. After that, several features are extracted from the preprocessed image. Finally, in the classification phase, several machine learning classifiers, such as K-nearest neighbour, support vector machines and Naïve Bayes classifiers are trained using the generated features in order to classify the images onto the respective classes.

**Fig. 8:**
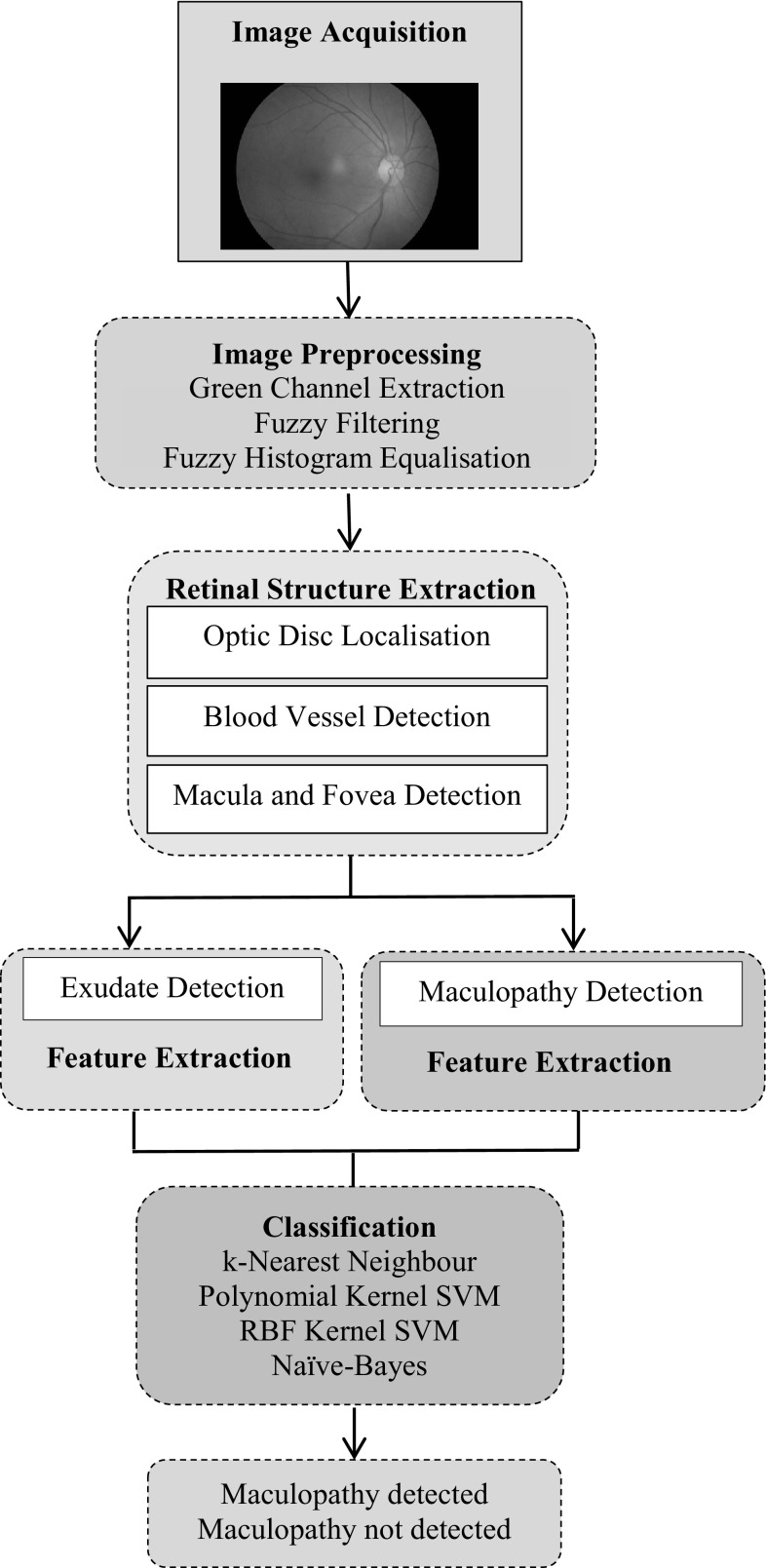
Block diagram of the proposed automatic detection of diabetic retinopathy and maculopathy using fuzzy image processing

Figure 8 shows the block diagram of the proposed system for automatic screening and classification of the diabetic retinopathy and maculopathy in retinal images using fuzzy image processing techniques. The individual stages are discussed in more detail in the following sections.

### Image preprocessing

**Fig. 9:**
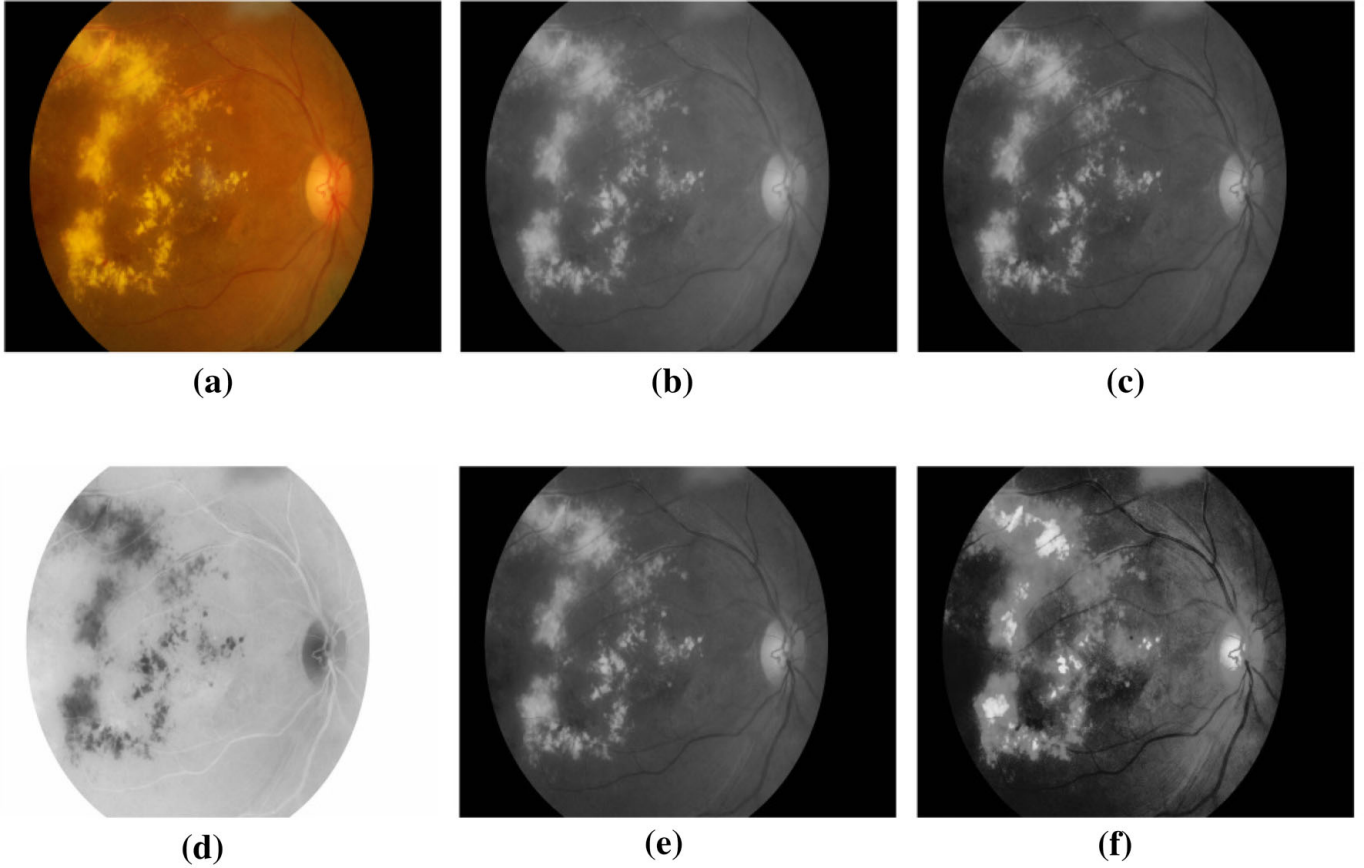
Preprocessing the output image. **a** Original image. **b** Greyscale conversion. **c** Green channel extraction. **d** Green channel complement. **e** Fuzzy filtering. **f** Fuzzy histogram equalisation

Image preprocessing takes place after the image acquisition process in order to improve the quality of the image. The present system utilises the following image preprocessing techniques: green channel extraction, fuzzy filtering and fuzzy histogram equalisation. Figure 9 shows the output image obtained after the preprocessing stages.

#### Green channel extraction

The first preprocessing technique extracts the green channel from the colour fundus image, which consists of the red, green and blue channels. In the previous system proposed by [6–8, 33], the colour fundus images are converted into a greyscale image. In the present system, the green channel extraction is used to investigate the difference and capability of the green channel format compared to the greyscale format and other image colour conversion formats. In most of the research in diabetic retinopathy screening carried out, the green channel is used for detecting the diabetic retinopathy lesions such as haemorrhages, vessels and microaneurysms. The red channel is somewhat saturated, while the green channel contains more structural information. Therefore, it is sensible to use the green channel for the segmentation or morphological operations, since most of the image processing tools work for greyscale only.

#### Fuzzy filtering

Image filtering needs to be implemented to improve the image quality or restore the digital image which tends to have a variety of noise types. The poor photo quality may be due to the equipment-related factors, such as dirty lens and dirty computer screen. In addition, distraction from surroundings, such as a too bright room, can be a factor of generating poor quality of fundus photographs. Therefore, the filtering process is necessary to overcome this problem and enables the image for further processing and grading task effectively. The proposed system implements the median filter with fuzzy techniques described by Toh et al. [10], called the Fuzzy Switching Median (FSM) filter. Although the proposed technique is not working well as an individual system variant in [7] for the microaneurysms detection, the technique has been working well in [8] for the diabetic retinopathy and maculopathy detection.

#### Fuzzy histogram equalisation

After filtering the image from noise, the third preprocessing technique, which is the fuzzy histogram equalisation, is performed on the images. Histogram equalisation’s role is to improve the image’s contrast. The colour fundus images are more challenging compared to the other modes of fundus photography examination, which are angiography and red-free. Therefore, the implementation of the histogram equalisation by employing fuzzy techniques helps to improve the contrast of the fundus images for better visualisation and detection. The technique called brightness preserving dynamic fuzzy histogram equalisation (BPDFHE) proposed by Sheet et al. [4] was found to work well on colour fundus images (see [6–8]) and has been chosen as a preprocessing technique in this proposed system as well.

### Retinal structure extraction

**Fig. 10:**
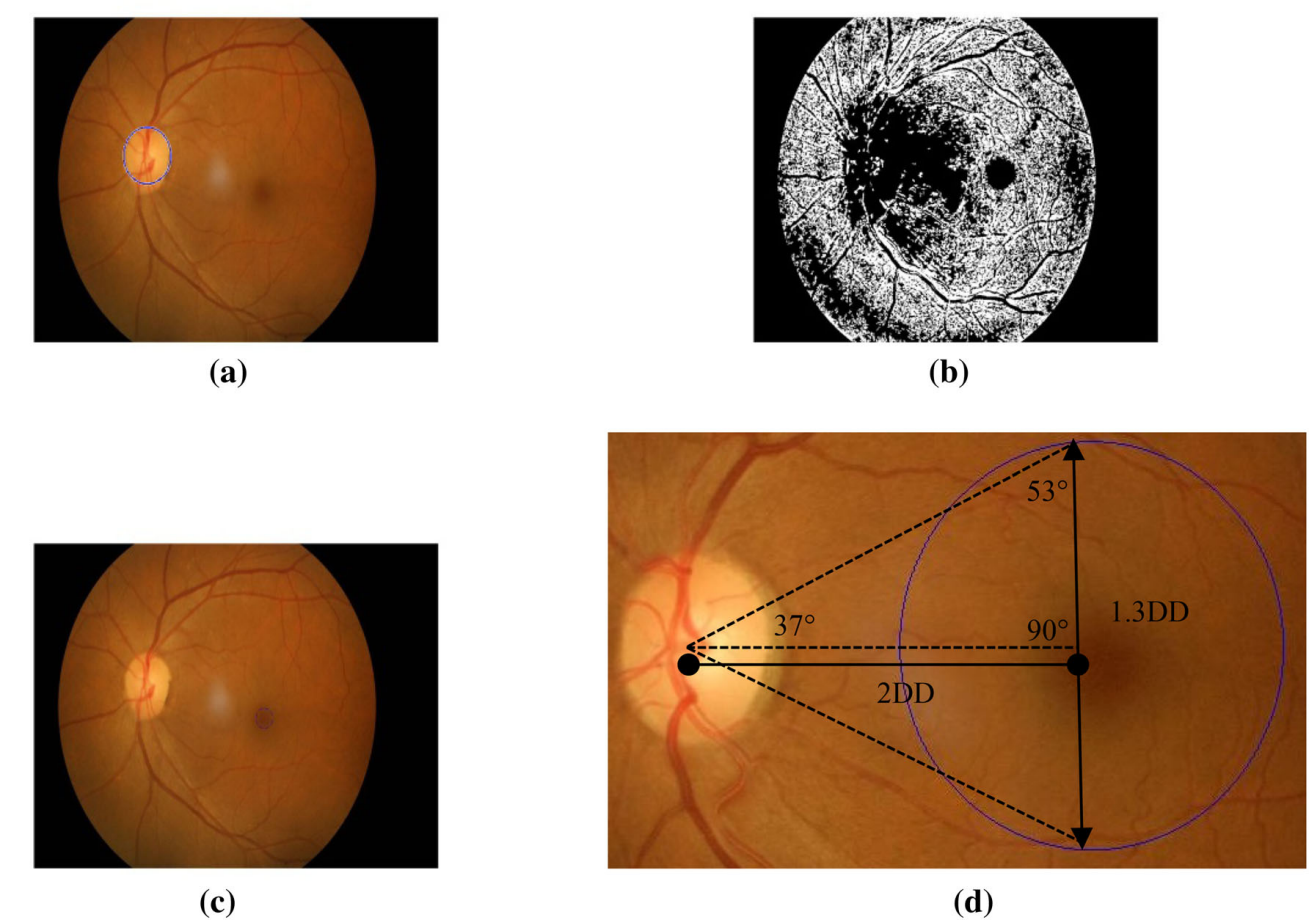
Extraction of retinal structures. **a** Optic disc detection. **b** Blood vessels segmentation. **c** Initial macula and fovea detection. **d** Proposed macula region

The extraction of retinal structures is helpful in eye diseases diagnosis, for improving the lesion detection results and the lesions grading. Therefore, the detection of retinal structures for diabetic retinopathy screening such as optic disc, blood vessels, macula and fovea are essential in order to produce a reliable screening system. Figure 10 shows the output image with retinal structures extraction.

#### Optic disc detection

The optic disc is the important retinal structure in the diabetic retinopathy screening. The optic disc has the largest high contrast among the circular shape areas. The location of the optic disc is of critical importance in retinal image analysis and is required as a prerequisite stage in detecting the lesions, such as in exudate detection. Therefore, the optic disc localisation and segmentation are both performed in the proposed system to improve the overall detection accuracy.

The technique used in the proposed system is based on circular Hough transform (CHT), to ensure more reliability of the optic disc detection task. Hough transform can be used to detect lines, circles or other parametric curves. In this case, the Hough transform for circles is used to locate the optic disc, due to the circle shape of the optic disc. Based on the specified radius range, the system finds the circles in fundus images using CHT with the use of *imfindcircles* function in Matlab. After finding the circles in the image based on the radius range, the function *viscircles* in Matlab is used to create a circle. The function draws circles with the specified centre and radius onto the current axes on the fundus images. The CHT technique has also been used for the detection of microaneurysms in [6–8], as this technique is capable well in detecting circular shapes. In addition to the CHT technique proposed, the polygonal Region of Interest (ROI) technique can be performed on the fundus image to detect the optic disc. A region of interest is a portion of an image for filtering purposes or for performing some other operations. In order to implement this method, the *roipoly* Matlab function is used, which aims to create an interactive polygon tool associated with the image displayed in the current image, called the target image. The function *roipoly* works by selecting vertices of the polygon and, as a result, it returns a binary image that can be used as mask for masked filtering. The function allows moving, deleting or resizing the polygon as well as moving, adding, deleting a vertex and also changing the colour of the polygon.

#### Blood vessels detection

After locating the optic disc, another important retinal structure, represented by the blood vessels, is extracted. There are several vessel extraction techniques, to name a few, morphological operations, Kirsch filter, Frangi filter, local entropy and entropic thresholding. For the proposed system, morphological operations are implemented for the extraction of the blood vessels. The operations start where the fuzzy histogram equalisation output image is opened using a disc-shaped structuring element. Next, the image background is removed and the image is thresholded to get the binary images containing the vessels. Finally, the morphological open is implemented by using the *bwareaopen* function to remove the small noise. In order to generate the elimination of vessels, the binary image can be subtracted from the Gaussian filtered image so that the final image has vessel-free candidates.

#### Macula and fovea detection

The detection of the optic disc is vital for the detection of the macula because the centre of the optic disc can be used for the macula detection. Macula appears as a dark region nearby the centre of the image and its location is at two disc diameter distance of the optic disc. The fovea is the centre of the macula. Once the macula is identified, it is simple to determine the fovea, as the centre of the macula. The macular region can be defined by using two clinical approaches. The first approach uses the Early Treatment Diabetic Retinopathy Study (ETDRS), which indicates the presence of clinical significant macular oedema (CSME) as any part located within one disc diameter of the centre of the fovea [53]. The second approach is based on the anatomical characteristics, where the macular region is about 5–5.5 mm in diameter.

In the proposed system, three methods for the macula detection are implemented. The first method is using the centre of the optic disc identified by the circular Hough transform. The macula is identified based on the distance specified from the optic disc. Once again, the circular Hough transform is used to create a circle for the macula region. We propose the detection of the macula region by an angle of 37° up and down from the horizontal line crossing the optic disc centre and with the centre of the macula region situated at two disc diameter (2DD) distance from the optic disc centre. We then crop a circle of 1.3 discs diameter (1.3DD) as the macula region where we try to find lesions. Figure 10d illustrates this process more clearly. The cropped macula region can be used to locate larger and appropriate lesion areas relevant to maculopathy. The *poly2mask* function can be used to get the region of interest of the macula region. In addition, the *imcrop* tool can also be used. However, cropping process has its limitation as it can be performed with rectangular shapes only, because images are represented as arrays in Matlab and arrays are required to be rectangular. The circular region of interest (ROI) cropping can be implemented with *roicirclecrop* function. The function crops the ROI in the form of circular shape and with black background based on the two points: the centre and the radius of the circle.

The second method is finding the centre of the image, where the macula is located in the centre of the retina for the images with the macula as centre view. The parameters of the circle, such as its location and radius size are initialised, followed by creating a circle mask in the image. The masking technique is performed later where the original image is masked with the circle to isolate the macula part of the image. Finally, the image masked with the circle is displayed as a macula output. In order to calculate the features from the output image, the current image is changed to a binary image. The white pixels around this region are presented as maculopathy.

The morphological operations also can be implemented as one of the ways to detect the macula. The top-hat transformation, which consists of morphological opening of the image and then subtracts the result from the original image operations, is implemented, followed by adding the original image to the top-hat-filtered image, and finally subtracting the bottom-hat-filtered image. As a result, the macula is detected.

### Exudate and maculopathy detection

In order to detect maculopathy, exudates first need to be detected in the macula region. One of the ways to detect the exudate lesions is by using the thresholding method. Thresholding is used to extract an object from its background by assigning an intensity value (threshold value) for each pixel, such as that pixel is either classified as an object pixel or a background pixel. In this case, the thresholding is used in the system development as the simplest form of segmentation to segment the fundus image into exudate region or background region. In the proposed system, the global thresholding or fixed thresholding is implemented, as it is one of the most popular types of thresholding methods. In global thresholding, the threshold value, *T*, is held constant throughout the image. Choosing a right threshold value is quite challenging and important to ensure that the value chosen is not too low or too high. In order to find a suitable threshold value for this system development, a software called ImageJ [54] is used (by using the thresholding slider bar from the Adjust Threshold menu). From our observations and the threshold value found through the searching process, it is concluded that a value of 135 is the most appropriate one to segment the exudate regions and the background in the fundus images obtained after the fuzzy histogram equalisation task. In addition to the global thresholding, fuzzy c-means (FCM) clustering can also be implemented in the system for detecting the exudates. Morphological operations can be used for the extraction of exudates too.

**Fig. 11:**
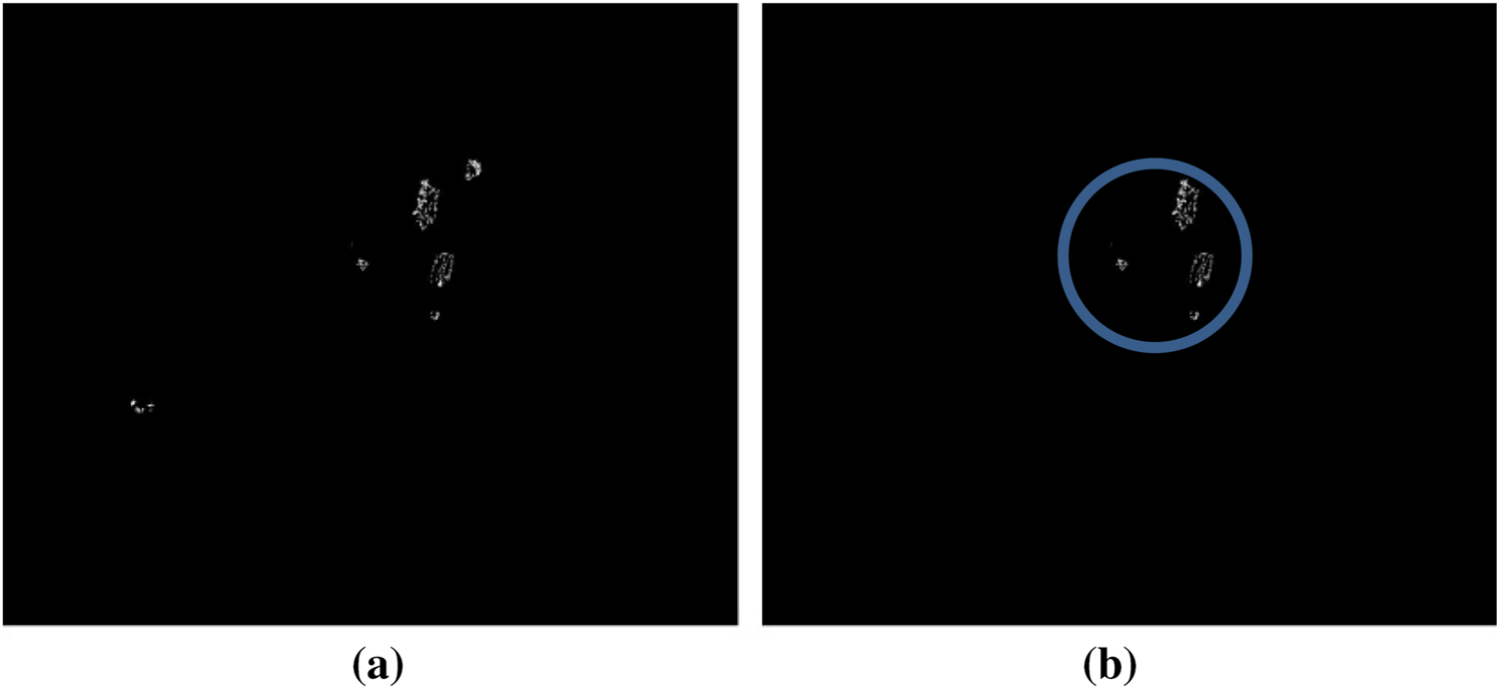
Exudates and maculopathy extraction output image. **a** Exudates detection. **b** Maculopathy detection

The image after the optic disc localisation and blood vessels segmentation is generated for the feature extraction task. We first convert it into a binary image in order to extract the exudates’ features. After the detection of the exudates, the maculopathy can be identified by cropping the area around the macula region. Figure 11a shows the exudates extraction with the optic disc overlay, while Fig. 11b shows the maculopathy output within the proposed macula region.

### Features extraction

After performing the preprocessing techniques, the retinal structure extraction and also the exudates detection, feature extraction takes place in order to obtain the features from the given images. There are three features selected from the exudates output image and macula image, respectively. They are the area of on pixels, the mean of on pixels and the standard deviation of the on pixels for the exudates region. In addition, there are another three features from the maculopathy region, i.e., the area of on pixels, the mean of on pixels and the standard deviation of on pixels for the maculopathy. The features between the exudates region and maculopathy region are compared. The value of the exudates area is higher compared to the area of the maculopathy value as the maculopathy is located around the macula, and the diameter of the macula is small.

### Classification

The six extracted features were used in the classification stage. As the system focuses on the detection of maculopathy, the classes from the developed dataset, as presented in Table 1 (in Sect. 3.2), have been categorised into two classes which are maculopathy detected or eye fundus with maculopathy (131 images) and maculopathy not detected or fundus without maculopathy (469 images). Several machine learning algorithms, such as the 1-nearest neighbour classifier, Naïve Bayes classifier, support vector machines (SVM) and radial basis function kernel SVM, have been selected to train and classify images into their respective classes. In the k-nearest neighbour classifier, the object is classified by a majority vote of its neighbours, with the object being assigned to the most common class among its *k*-nearest neighbours. A support vector machine performs the classification by constructing an *N*-dimensional hyperplane that optimally separates the data into two categories. The radial basis function kernel is used to transform the data into a higher dimensional space in order to be able to perform the separation in the non-linear region, while Naïve Bayes classifier is based on the Bayes’ theorem with independence assumptions between predictors. The Naive Bayes can outperform more sophisticated classification methods, despite its simplicity.

### System results

**Fig. 12:**
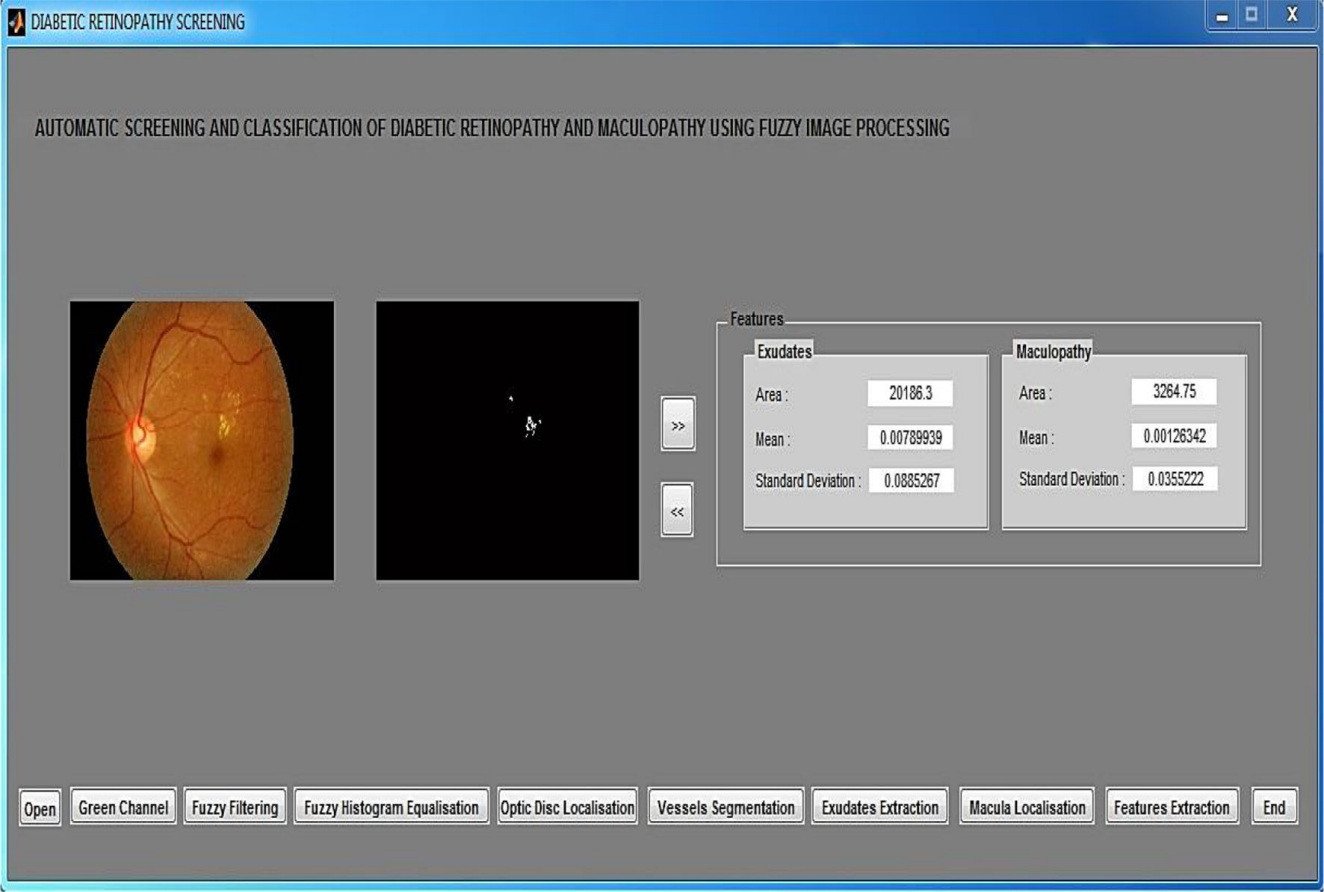
Snapshot of the proposed system user interface

Figure 12 shows the user interface snapshot of the proposed system. The results are shown in Table 5. The confusion matrix, the sensitivity, the specificity and the accuracy of the individual classifiers are presented. Since the maculopathy detected class (131 images) is imbalanced compared to the maculopathy not detected class (469 images), the maculopathy detected class was oversampled several times. As a result, a total of 990 images, which consist of 469 images from the maculopathy not detected class and 521 images from the maculopathy detected class are involved in the final classification stage. The new dataset is split randomly into 90 % for training and the remaining 10 % for testing purpose. The process is repeated ten times in a cross-validation procedure to generate unbiased results. The results are averaged over ten runs for each of the classifiers. The experimental results show that the four classifiers are able to identify well both categories, and particularly the k-nearest neighbour and radial basis function kernel support vector machine classifiers work well. The detection of exudates first is important because the presence of exudates will determine the presence of the maculopathy or not. Based on the presented results, it can be seen that the sensitivity value is a little bit lower than the specificity. This is due to the fact that the total number for maculopathy detected (abnormal) cases is lower than the total number for maculopathy not detected cases (normal), which was explained above. The other reason may be the capability of global thresholding technique which was used for the detection of the exudates; the proposed threshold value may be quite high and, as a result, some of the exudates were not detected by the proposed technique.

**Table 5:** Average results when using the four classifiers

	k-nearest neighbour	Polynomial Kernel SVM	RBF Kernel SVM	Naïve Bayes
Misclassification error	0.0700	0.3000	0.0700	0.2500
Accuracy	0.9300	0.7000	0.9300	0.7500
Specificity	1.0000	0.9787	0.9362	0.9149
Sensitivity	0.8679	0.4528	0.9245	0.6038

## Conclusions and future work

An automatic system for the combined detection of diabetic retinopathy and diabetic maculopathy using fuzzy image processing techniques has been created. The system proposes a novel combination of fuzzy image preprocessing techniques including the retinal structures localisation, (i.e. for the optic disc, the blood vessels, the macula and the fovea), followed by the feature extraction and, finally, the classification with some machine learning algorithms. The system can be enhanced by implementing other fuzzy image preprocessing techniques to fully investigate the capabilities of a variety of fuzzy techniques in this application. Fuzzy circular Hough transform, as an alternative technique to detect the optic disc, will be considered in our future work. Since the detection of maculopathy presence depends on the exudate detection performance, more precise exudate detection methods need to be developed in the future as well. As a conclusion, employing fuzzy image processing together with the retinal structure extraction in diabetic retinopathy screening can help produce a more reliable screening system. The paper is not only proposing an automatic detection for diabetic retinopathy and maculopathy, but also introducing a novel dataset which would be useful to researchers and practitioners working in the retinal imaging area, especially in the diabetic retinopathy screening field. The online dataset can be made more widely accessible by integrating the dataset with other popular databases, such as the UCI machine learning repository or others.
